# A Framework for Addressing Implementation Gap in Global Drowning Prevention Interventions: Experiences from Bangladesh

**Published:** 2014-12

**Authors:** Adnan A. Hyder, Olakunle Alonge, Siran He, Shirin Wadhwaniya, Fazlur Rahman, Aminur Rahman, Shams El Arifeen

**Affiliations:** ^1^Johns Hopkins International Injury Research Unit, Department of International Health, Johns Hopkins Bloomberg School of Public Health, 615 N. Wolfe Street, Baltimore, MD 21205, USA; ^2^Centre for Injury Prevention and Research, Bangladesh (CIPRB), House 162B, Road 23, New DOHS, Mohakhali, Dhaka 1206, Bangladesh; ^3^Centre for Child and Adolescent Health, icddr,b, 68 Shaheed Tajuddin Ahmed Sarani, Mohakhali, Dhaka 1212, Bangladesh

**Keywords:** Childhood injury, Drowning, Implementation framework, Implementation science, Interventions, Low- and middle-income countries, Prevention, Bangladesh, South Asia

## Abstract

Drowning is the commonest cause of injury-related deaths among under-five children worldwide, and 95% of deaths occur in low- and middle-income countries (LMICs) where there are implementation gaps in the drowning prevention interventions. This article reviews common interventions for drowning prevention, introduces a framework for effective implementation of such interventions, and describes the Saving of Lives from Drowning (SoLiD) Project in Bangladesh, which is based on this framework. A review of the systematic reviews on drowning interventions was conducted, and original research articles were pulled and summarized into broad prevention categories. The implementation framework builds upon two existing frameworks and categorizes the implementing process for drowning prevention interventions into four phases: planning, engaging, executing, and evaluating. Eleven key characteristics are mapped in these phases. The framework was applied to drowning prevention projects that have been undertaken in some LMICs to illustrate major challenges to implementation. The implementation process for the SoLiD Project in Bangladesh is used as an example to illustrate the practical utilization of the framework. Drowning interventions, such as pool fencing and covering of water hazards, are effective in high-income countries; however, most of these interventions have not been tested in LMICs. The critical components of the four phases of implementing drowning prevention interventions may include: (i) planning—global funding, political will, scale, sustainability, and capacity building; (ii) engaging—coordination, involvement of appropriate individuals; (iii) executing—focused action, multisectoral actions, quality of execution; and (iv) evaluating—rigorous monitoring and evaluation. Some of the challenges to implementing drowning prevention interventions in LMICs include insufficient funds, lack of technical capacity, and limited coordination among stakeholders and implementers. The SoLiD Project in Bangladesh incorporates some of these lessons and key features of the proposed framework. The framework presented in this paper was a useful tool for implementing drowning prevention interventions in Bangladesh and may be useful for adaptation in drowning and injury prevention programmes of other LMIC settings.

## INTRODUCTION

Globally, the total deaths caused by injuries increased from 4 million in 1990 to 5 million in 2011 (1). According to the World Health Organization (WHO), more than 359,000 deaths in 2011 were due to drowning (1). Thirty-six percent of these deaths occurred among individuals aged below 15 years; children aged 1-4 year(s) accounted for 18% of all drowning-related deaths (1). Drowning is the second leading cause of childhood injury-related deaths worldwide and is the commonest cause of injury-related deaths among under-five children (1,2).

The 2011 WHO estimates show that the burden of drowning is disproportionately borne by populations in low- and middle-income countries (LMICs) (1). Analyses of community-based surveillance data from Southeast Asian countries show that those aged 1-4 year(s) are at the highest risk of drowning (3). In Bangladesh, drowning accounts for 42% of all deaths in children aged 1-4 year(s) (4). These deaths occur particularly in rural areas of the country; 75% of deaths occur in natural water bodies less than 20 metres from the home (5,6). Other risk factors for childhood drowning in Bangladesh include inadequate supervision, male gender, and the monsoon/rainy season (April-September) (7,8). Indeed, lack of direct supervision often leads to childhood drowning worldwide and has been associated with 70% of drowning-related deaths among children in Bangladesh (9).

Several attempts have been made in the past to tackle the high rate of childhood drowning in Bangladesh through prevention (10,11). Two supervisory tools—door barriers and playpens—were pilot-tested in rural areas of the country to prevent childhood drowning (10). While both tools improved supervision, playpens found a greater acceptance among the caregivers (10). Another study explored the option of a crèche which provides a suitable environment for primary caregivers to place children in during busy hours for the family. The study reported a drastic drop in drowning rate and risk among 1 to 5 year(s) old children who participated in the programme, with children being 72% less likely to drown, compared to those who did not participate (11). While these two interventions hold significant promise in reducing the burden of childhood drowning in Bangladesh, these have not been implemented at scale. In fact, it has been proposed that funding for a large-scale drowning prevention intervention study focusing on under-5 children in LMICs may be an appropriate research investment (5).

In a previous paper (Hyder *et al*. 2014), we described the protocol for a implementation study, the Saving of Children's Lives from Drowning (SoLiD) Project, to implement the playpen and crèche interventions at a large-scale in Bangladesh (12). The overall goal of this paper is to present a framework for implementing drowning prevention interventions in resource-limited settings (13,14). This framework was used in developing the SoLiD Project. To provide some background on the discussion of the framework and its application to SoLiD, a brief review of selected potential intervention options for addressing the burden of drowning in LMICs is also presented. The hope is that this paper will further the dialogue on how to close the implementation gap for childhood drowning prevention interventions in LMICs and provide guidance on initial steps for implementing these interventions in resource-limited settings (12).

## SELECTED INTERVENTION OPTIONS FOR CHILDHOOD DROWNING PREVENTION AND THEIR RELEVANCE IN LMICs

A review of the systematic reviews on drowning prevention interventions was conducted, and original research articles were pulled and summarized into broad prevention categories. The initial review was based on the WHO World Report on Child Injury Prevention (WRCIP) (7). The WRCIP report comprises a systematic review of interventions for major child injury-related death mechanisms, including drowning. Drowning interventions identified in the report were summarized under the following broad prevention categories: engineering measures, environmental measures, legislation and standards, education and skills, and drowning management (7). Additionally, a review of the Cochrane Database for systematic reviews on drowning prevention interventions was conducted to identify other interventions that may not have been covered in the WRCIP report. A total of 30 articles—both original research articles and systematic reviews—were identified that contained information on drowning prevention interventions. We included 13 articles based on the type of study (randomized controlled trials, case-control studies, and observational studies) and on the age-group these focused on (generally defined as ‘children’, with varying age definitions in different studies).

Table 1 presents an overview of all the drowning intervention options considered. The broad categories for classifying the intervention options are defined in this paper as follows:

‘Engineering measures’ involve complete removal of hazards by active and passive strategies to creating an injury-free infrastructure (7). ‘Environmental methods’ aim at modifying an existing injury-prone environment to eliminate risks. Indeed, environmental measures cannot be fully implemented without corresponding ‘legislation and regulation’. For instance, pool fencing would not have been successful in many industrialized countries without enforcement of pool fencing laws. ‘Education and skills’ refer to a range of efforts aimed at increasing knowledge of water safety, adoption of safer behaviours and improving survival in an emergency (10,11,15-19). ‘Drowning management’ is confined to cardiopulmonary resuscitation (including oxygen therapy and mechanical ventilation) and rescue methods (such as utilization of an intermediary object and a neck floatation device that are important in near-drowning events) (20).

**Table 1.** Examples of interventions studied for childhood drowning prevention

Type of intervention	Intervention strategy (study type)	Lead author (publication year)	Location	Age-group	Main findings
Environmental measures	Covering water sources (case-control)	Celis (1997)	Mexico	1-4 year(s)	Any uncovered water body in and around home environment is a risk factor. Drowning risk (OR=6.8) compares homes with wells with those who are without wells
	Pool fencing (review article on 3 case-control studies)	Thompson (2000)	Australia and New Zealand	<14 years	Drowning mortality reduction: OR range (0.17-0.29)
Legislation and standards	Pool fencing laws and their enforcement (national telephone survey)	Logan (1998)	USA	<5 years	19% of pool-related drowning events could have been prevented by proper fencing of all residential pools
	Personal floating devices (observational)	Wintemute (2013)	California, USA	<14 years	No evidence for effectiveness in drowning prevention
	Influence of alcohol on drowning (case study)	Schyllander (2013)	Sweden	0-17 years	Use of alcohol by older victims is a risk factor
Developing education and skills	Swimming programmes (review)	Brenner (2003)	Canada, Australia, USA, etc.	<16 years	Protective relationship between increased swimming ability and the risk of drowning has never been demonstrated
	Training of lifeguards (commentary)	Schwebel (2010)	USA	0-18 year(s)	Regular training and practice of lifeguard is essential
	Water safety education among children (pre-post)	Solomon (2012)	Spain	5-12 years	Students’ water safety knowledge significantly increased but no data linked to the reduction of drowning
	Water safety education to parents/caregivers (pre-post)	Moran (2006)	New Zealand	Parents of 2-4 years old children	Statistically significant improvement in parental understanding of water safety
	Supervision by parent- or caregiver-supervised bath (observational)	Cass (1996)	Australia	0-14 year(s)	Drowning in bath did not decrease from 1990 to 1995
	Supervision by parent or caregiver—door barriers and playpens (qualitative)	Challaghan (2010)	Matlab, Bangladesh	6-54 months	High compliance rate; positive potential of playpen to improve parental supervision practices
	Supervision—crèche (cost-effectiveness analysis)	Rahman (2012)	Bangladesh	Crèche: 1-5 year(s)	Crèche: US$ 812 per DALY averted
				SwimSafe: 4-12 years	SwimSafe: US$ 85 per DALY averted
Managing drowning	CPR training (report)	CDC (2012)	United States	1-4 year(s)	All caregivers and supervisors should have training in cardiopulmonary resuscitation

As shown in Table 1, many of these examples are from high-income countries (HICs). While some have been shown to be effective (for example, covering of water bodies and pool fencing) in the HIC settings, others (such as water safety education, swimming instructions, and personal floating devices) are yet to be studied comprehensively (5,15,17,21-25). Unfortunately, interventions developed in the HIC settings may not be directly applicable to the LMIC settings. For example, while pool fencing is ideal for the HIC settings where most drowning incidences occur in swimming pools, it may not be a realistic option for LMICs because most drowning incidences take place in natural water bodies of rural areas (5). Personal floating devices are reported to contribute positively to drowning prevention; yet no definite evidence has been obtained to prove its effectiveness in both HIC and LMIC settings. Also, it is impossible to have all children carrying lifejackets around at all times, given that the majority of childhood drowning incidences in LMICs occur within and around the home environment (25). Whereas swimming instruction has been extensively studied and implemented in many HICs, the protective impact of swimming lessons on childhood drowning prevention has not been conclusively established for under-five children in LMICs (15). Moreover, in an LMIC such as Bangladesh where the peak age for drowning is 1 year, such young children are not developmentally ready to acquire swimming skills required for survival (15). Also, lifeguards cannot be presented as a ubiquitous solution in most LMIC settings, given the cost and type of water bodies where most drowning cases occur. While safety education among school-aged children has been reported to enhance the knowledge of children regarding water safety, no data have linked the effort of safety education to the reduction of drowning incidences (17).

This state of knowledge reflects the challenge of testing feasible and appropriate interventions for effective large-scale drowning prevention in LMICs, thus necessitating the development of a framework that assists in the implementation process of such activities.

## FRAMEWORK FOR IMPLEMENTATION OF DROWNING INTERVENTIONS

The proposed framework for effective implementation of drowning prevention interventions is based on a literature review of frameworks for implementing large-scale injury programmes. It builds mainly upon two existing frameworks—from the fields of road traffic injury prevention by Hyder (the first author of this article) and others, 2012 and implementation science by Damschroder *et al.* 2009 (13,14). Relevant constructs from these two frameworks were extracted to develop a modified framework that can be specifically applied to implementation of drowning prevention interventions in LMICs. The modified framework was then applied to drowning prevention projects previously executed in some LMICs to illustrate major challenges to implementation of drowning interventions in these settings.

Effective implementation is an indispensable part of any successful intervention rollout. Potential characteristics of an effective implementation process have been proposed in the literature, especially around implementation science (13,14). Damschroder *et al.* 2009 developed a consolidated framework with five domains, namely intervention characteristics, outer settings, inner settings, characteristics of the individuals involved, and the process of implementation (14). The ‘implementation process’ they described included four phases: planning, engaging, executing, and evaluating. Similarly, major characteristics of an effective response to the implementation gap in injury prevention have been described by the first author of this article, ranging from global funding and political will to sustainability and rigorous evaluation (13). Eleven of these characteristics were mapped in the four phases of the implementation process to develop the proposed framework for implementation of drowning interventions as presented in this paper (Table 2). To illustrate the utility of this framework, selected literature of projects implementing drowning prevention interventions in LMICs were reviewed using this framework to reveal the common challenges they faced during the implementation process (Table 2) (11,26-28).

‘Planning’ (the first phase of the implementation process) refers to the process of identifying and organizing resources for an impending action; it entails outlining comprehensive activities that will ensure effective and sustainable uptake of the intervention (29). The specific planning strategies may vary between projects and stakeholders and can be tailored according to local needs and resources. The second phase ‘engaging’ is the act of actively soliciting the involvement of stakeholders─relevant institutions and individuals─in the implementation process (14). The third phase ‘executing’ is the process of implementing the project as planned (14). Lastly, the ‘evaluation’ phase involves providing comprehensive feedback on the project implementation, both qualitatively and quantitatively, to stakeholders (14).

**Table 2.** Framework for implementation of drowning interventions and practical challenges in LMICs

Phases of implementation process (12)	Key characteristics (12,14)	Exemplary challenges in LMICs* (10,25-27).
Planning	1. Global funding 2. Political will 3. Scale 4. Sustainability 5. Building capacity	Need additional funding from governments, donors, or other sources (Bangladesh, Cambodia, China, Philippines, Thailand, Viet Nam) No policies/ordinance/safety measures for drowning prevention (Philippines) Lack of surveillance and reporting system (Bangladesh, Cambodia, China, Philippines, Thailand, Viet Nam) Lack of skills/skilled workers/trained community personnel/rescuers (Philippines, Viet Nam) Financial resources from local organizations were not mobilized to support injury prevention (Viet Nam) The programme activities were not yet integrated effectively into other local activities (Bangladesh, Cambodia, China, Philippines, Thailand, Viet Nam) Lack of experience and capacity (Bangladesh, Cambodia, China, Philippines, Thailand, Viet Nam) Lack of financial and social capital to promote a culture of water safety (Bangladesh, Cambodia, China, Philippines, Thailand, Viet Nam) High illiteracy (Bangladesh, Cambodia, China, Philippines, Thailand, Viet Nam) Challenges in scaling up a package of effective drowning intervention (Bangladesh, Cambodia, China, Philippines, Thailand, Viet Nam)
Engaging	6. Coordination across actors 7. Involving appropriate individuals	Inadequate cooperation from different sectors (Philippines) Inadequate role assignment for various personnel/stakeholders (Philippines, Viet Nam)
Executing	8. Focused action 9. Multisectoral action 10. Quality of execution	Societal barriers (Bangladesh, Cambodia, China, Philippines, Thailand, Viet Nam): In rural area, water and other environmental hazards are ubiquitous Building codes and zoning ordinance are lacking or unenforced High levels of illiteracy across large segments of the population Parents rely on older children to supervise younger ones Large family-size Insufficient community response Very few social services available for emergency medicine
Evaluation	11. Rigorous monitoring and evaluation	Supervising and monitoring activities are not up to required standard (Viet Nam)

*Practical challenges are categorized by phase, and not necessarily matched by each characteristic; LMIC=Low- and middle-income country

### Phase 1: Planning

During the ‘planning’ phase, several studies in LMICs mentioned insufficient financial investment as a major factor that significantly hampers successful implementation of any drowning prevention programme. Obtaining additional funding from external donors, stimulating and sustaining adequate budget allocation within the health sector, and sufficient mobilization of financial resources from local organizations were all tangible challenges faced by implementers (11,26-28). Lack of political will or government buy-in was also a common problem; lack of technical capacity at local and national levels was also commonly cited (26-28). Capacity building is crucial to the high-level performance required for effective implementation and often requires special training for intervention workers and non-project personnel in government and recipient communities. Furthermore, it is important to consider carefully if the intervention is scalable and may be implemented at a scale that will enable researchers to evaluate it. Long-term sustainability of the implementation process should also be considered.

### Phase 2: Engaging

During the ‘engaging’ phase, coordinating across sectors and involving appropriate individuals are of primary concern. For instance, inadequate cooperation from health and allied sectors—combined with improper role assignment for various stakeholders–significantly hampered the implementation of a drowning prevention package in the Philippines (26). In addition, influential individuals, such as opinion leaders, locally-supported champions as well as external consultants should be recruited to play key roles in the implementation process to increase acceptability of the intervention (14).

### Phase 3: Executing

The executing phase requires focused action, close collaboration across multiple sectors, and tracking implementation fidelity. Focused action refers to the provision of the intervention to the most at-risk groups in the most efficient way in an area with high burden of drowning. For example, to prevent drowning in Bangladesh, targeting 1 to 4 year(s) old children in areas with the highest burden (rural Bangladesh) is expected to be most effective for the implementation process. Multisectoral collaborations that are initiated during the engaging phase become more important during the executing phase and are further extended to coordinate implementation efforts among multiple partners, enhance programme ownership, promote public-private partnerships, and encourage programme sustainability. During this phase, ensuring adequate programme fidelity, intensity, and a reasonable timeline for task completion (factors which reflect quality of execution) is the key (Table 2).

### Phase 4: Evaluating

The last phase occurs simultaneously with the executing phase, and it involves rigorous monitoring and evaluation of both intervention and implementation process. This requires careful consideration of evaluation design, balance between rigor and real-world context, and defining an analytic strategy (Table 2).

The Saving of Children's Lives from Drowning Project in Bangladesh, a large-scale drowning prevention study, is narrated below to illustrate the practical utilization of this modified framework.

## APPLICATION OF FRAMEWORK FOR EFFECTIVE IMPLEMENTATION: A PRACTICAL EXAMPLE

The Saving of Lives from Drowning (SoLiD) Project is a response to the high burden of childhood drowning in Bangladesh and, to our knowledge, one of the largest childhood drowning prevention projects ever undertaken in LMICs (12). It is a collaborative effort by two research institutions based in Bangladesh—icddr,b and the Centre for Injury Prevention Research, Bangladesh (CIPRB)—and the Johns Hopkins International Injury Research Unit (JH-IIRU) at Johns Hopkins Bloomberg School of Public Health, based in Baltimore. It is sponsored by the Bloomberg Philanthropies (Table 3). The Project will target an estimated population of 1.3 million in rural Bangladesh and will provide two drowning prevention interventions for under-five children. The interventions—the playpen and crèche (which are described in detail below)—have been previously tested in rural Bangladesh (9-11), and the effectiveness and cost-effectiveness of the crèche for child drowning prevention have been demonstrated in this setting (11). Both of these interventions will be combined with family education and community awareness of injury prevention strategies. Ethical approval for the Project was secured from the Institutional Review Board at Johns Hopkins Bloomberg School of Public Health (JHSPH), the Research Ethics Committee of icddr,b, and the Ethics Review Committee of CIPRB.

**Table 3.** A collaborative implementation research triumvirate

Organization	Institution type	Description	Primary role	Coverage
Johns Hopkins International Injury Research Unit, Johns Hopkins Bloomberg School of Public Health, USA	University research centre	Injury-related research, collaboration and training; World Health Organization Collaboration Centre for Injury, Violence and Accident Prevention, Bangladesh	Study design; Monitoring and evaluation; Technical support	All sites
International Centre for Diarrhoeal Disease Research, Bangladesh	Research Institution	Research, training, and extended activities; Maintaining surveillance system since 1966 in Matlab	Intervention implementation; Baseline survey and surveillance;	Matlab North, Matlab South, and Daudkandi
Centre for Injury Prevention and Research, Bangladesh	Non-governmental organization	Injury prevention and research Innovative community programmes Training	Intervention implementation; Baseline survey and surveillance;	Raiganj, Sherpur Sadar, and Monohordi

Table 4 shows how the 11 characteristics outlined in the modified framework were implemented within the SoLiD Project to address some of the challenges identified from previous drowning prevention projects from other LMICs. The characteristics are not listed in their original order but described, along with the introduction of the SoLiD Project below.

Securing the support of a global private donor—Bloomberg Philanthropies (Characteristics 1: Global funding)—and coordination among implementing partners within and outside Bangladesh, icddr,b, CIPRB, and JH-IIRU [Characteristics 6: Coordination across actors; Characteristics 7: Involving appropriate individuals; Characteristics 9: Multisectoral action (Table 4)] are crucial for channeling and optimizing resources—financial, technical, and others—to address drowning of under-five children in rural Bangladesh.

### Study participants

**Figure 1.**
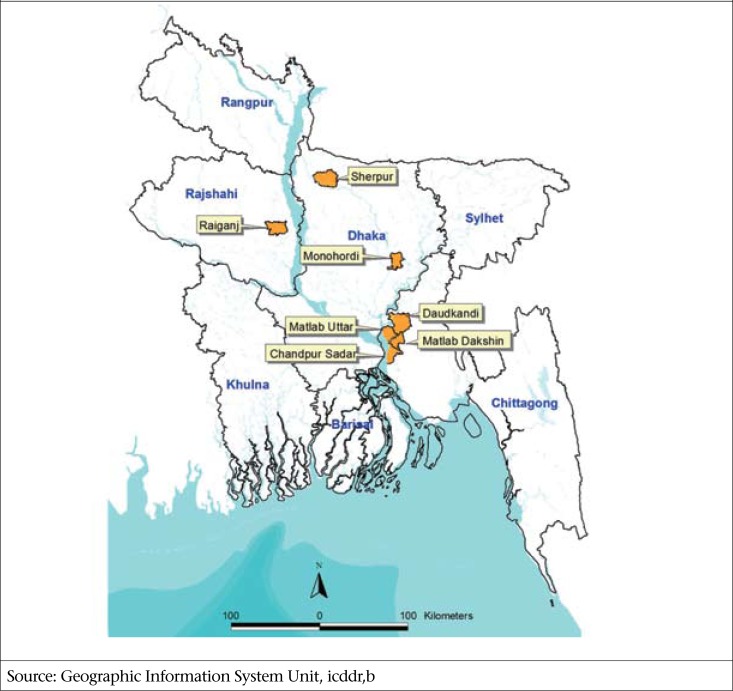
Map of Saving of Children's Lives from Drowning (SoLiD) study sites in Bangladesh

The SoLiD study will be implemented for under-five children in seven purposively selected subdistricts of Bangladesh—Matlab South, Matlab North, Daudkandi, Chandpur Sadar, Raiganj, Sherpur Sadar, and Monohordi (Figure 1). These targeted regions have some of the highest childhood drowning risks in Bangladesh (Characteristics 8. Focused action, Table 4). These subdistricts were selected also because some of their populations are already covered under routine demographic and injury surveillance carried out by the implementing partners; this would facilitate participation in and evaluation of the study (Characteristics 11. Evaluation, Table 4). All children of both sexes within the age of 9 and 36 months residing in these selected subdistricts would be recruited to participate in the study. The Project will cover an estimated 1.3 million people and deliver the package to an estimated 80,000 children (Characteristics 3. Scale, Table 4). This universal coverage of the study benefits is recommended by WHO (7).

**Table 4.** Application of the implementation framework to Saving of Lives from Drowning Project, Bangladesh

Implementation process	Characteristics	SoLiD Project feature
Planning	1. Global funding	Bloomberg Philanthropies
	2. Political will	Conducted series of advocacy meetings with local political leaders
	3. Scale	7 sites throughout Bangladesh; 80,000 children; 1.3 million people
	4. Sustainability	Local hiring of project staff; local production of intervention tools; community ownership/involvement
	5. Building capacity	High technical capacity identified among local implementers; regular professional training Project staff-specialized training Good human resources
Engaging	6. Coordination across actors	International research institutions and non-governmental organizations Coordination among local government officials, village representatives, and project staff
	7. Involving appropriate individuals	Central-level—qualified researchers; local level—formally-appointed internal implementation leaders; local champions nominated by village committees selected as childcare workers
Executing	8. Focused action	Targeting regions with the highest childhood drowning risk [children aged 1-4 year(s)]
	9. Multisectoral action	Multisectorial action inherent in design
		The intervention is a modification of previously pilot-tested effective strategies appropriate for local settings
	10. Quality of execution	Using established standards and formative research to inform intervention design, preparing standard operating procedures during the planning phase to ensure high degree of fidelity, appropriate intensity, timeliness of task completion, adequate engagement, and pay for performance on the quality of execution
Evaluation	11. Rigorous monitoring and evaluation	Quantitative and qualitative evaluation; regular progress reporting and monitoring

### Study design

As described previously (Hyder *et al.* 2014), the playpen is a rigid four-sided enclosure with slats and a firm base that serves as a barrier between its occupant (toddler) and the external physical environment (12). It serves as a primary barrier and limits exposure of a child to the risk of drowning when the caregiver is busy with household activities. The playpens will be used in the SoLiD Project and will be manufactured in Bangladesh from locally-available wood and safe plastic (Characteristics 4. Sustainability, Table 4). The playpens were designed based on guidelines from the US Consumer Protection and Safety Commission (CPSC) and prior formative research that was conducted in Matlab, Bangladesh (10,30) (Characteristics 10, Quality of execution, Table 4)

The crèche is a daycare centre that operates between 9:00 am and 1:00 pm, the peak period in Bangladesh for household chores and when a child is most at risk of drowning (3). Activities in the crèche include learning language and numbers, reading, dancing, drawing, and other activities that stimulate cognitive and motor skills; children would also learn about local cultural norms, health, hygiene, and injury prevention through singing, role-playing, and storytelling. In each crèche, two trained childcare workers will supervise 24-30 children. The crèche was first used as a childhood drowning prevention tool by CIPRB (and was called ‘Anchal’) (11).

An initial baseline census would be carried out in the 7 selected subdistricts. Thereafter, a demographic and injury surveillance system will be maintained for the entire population (of approximately 1.3 million) to monitor population changes, injury outcomes, and participation in the interventions during the project life. A surveillance visit will be conducted every 2-4 months (Characteristics 10. Quality of execution, Table 4). In addition, intervention workers will visit households with enrolled children to assess compliance and satisfaction with the interventions every 2 months for the playpen intervention and every 4-6 months for the crèche intervention (Characteristics 11. Rigorous monitoring and evaluation, Table 4).

The SoLiD Project is using community workers from local and regional areas. Identifying project staff with good qualifications and experience from the local communities is important for building community ownership (Characteristics 4. Sustainability, Table 4) and providing opportunity for professional training and re-training of local change agents by the SoLiD Project (Characteristics 5. Building capacity, Table 4). And because of the scale of this project, the impact of these individuals in advancing public health in these communities would likely extend beyond the injury field to other public health areas.

At the crèche facility level, project supervisors will oversee the activities of the trained childcare workers and monitor the utilization and quality of services being provided at the daycare centre. The supervisors will, in turn, report to monitoring officers monthly, and these officers will facilitate audit meetings as needed and maintain a continuous training curriculum for the childcare workers. Community oversight will be provided through village injury prevention committees that will be formulated prior to the selection of venues for the crèches. These committees will nominate local champions to serve as childcare workers, select the crèche venues and will meet regularly to resolve issues that may affect the operations of the crèche, generate awareness of the interventions among potential beneficiaries, and help raise local resources to meet any urgent needs that may arise. (Characteristics 4. Sustainability; Characteristic 7. Involving appropriate individuals; Characteristics 10. Quality of execution, Table 4). In addition, series of advocacy meetings with local political leaders will be conducted to inform the authorities as well as gain political buy-in from them prior to the project start up. (Characteristics 2. Political will, Table 4).

**Figure 2.**
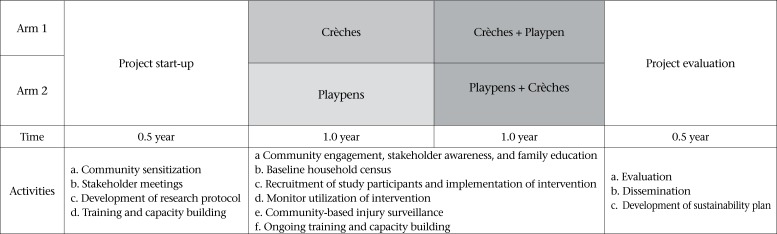
Saving of Children's Lives from Drowning (SoLiD) study: design and timeline

A pre-post, quasi-experimental study design will be employed to compare drowning rates before and after the interventions in the participating communities (Figure 2). The study population will serve as their own historical control, and comparison data will be gathered through a 5-year recall, collected during the baseline survey and through the use of panel historical data available from local collaborators (31,32). The intervention package will be implemented over a period of two years. In the first year, the playpen intervention (along with educational activities) will be implemented among enrolled children in four subdistricts with icddr,b interventions (Matlab North, Matlab South, Daudkandi, Chandpur Sadar) while the crèche intervention will be implemented in three CIPRB-managed subdistricts (Raiganj, Sherpur Sadar, and Monohordi). At the beginning of the second year, all eligible children in 7 study areas will be offered the other intervention.

### Assuring implementation quality

A strategic strength of the SoLiD Project is the availability of technical capacity to implement the interventions at scale. The two implementing partners─icddr,b and CIPRB─are organizations with extensive operations and research experience in Bangladesh. Both organizations will conduct regular coordination meetings and their staff will receive further training adapted specifically for the SoLiD Project (Characteristics 10. Quality of execution, Table 4). Furthermore, all standard operating procedures for major activities, including baseline data collection, rollout of the intervention package, and setting up surveillance and data systems, were developed during the planning phase to ensure a consistency of approach during executing phase (Characteristics 10. Quality of execution, Table 4). The standard operating procedures contain the core elements essential for each project activity. Each implementer is allowed the flexibility to execute these activities without compromising the core elements, thus accomplishing the twin goals of adaptation and fidelity. In addition, the disbursement of funds to partners is tied to timely delivery of core project activities (Characteristics 10. Quality of execution, Table 4).

## DISCUSSION

The implementation gap surrounding childhood drowning prevention interventions is a challenge for global health, especially as effective intervention strategies available in HICs are generally not directly applicable to LMIC settings. Evidence points to the effectiveness of child supervision strategies in drowning prevention, and yet there is lack of large-scale implementation studies that validate the effectiveness of these strategies. The SoLiD Project targets an area with one of the highest burden of drowning in the world and is an opportunity to fill this global knowledge gap (8).

Conducting large-scale implementation research projects requires considerable time and effort in any setting, including LMICs, like Bangladesh. This is why the framework for implementation of drowning interventions was designed and applied to ensure effective rollout of the SoLiD intervention package and to learn lessons from other similar work. Despite such efforts, there are aspects of the implementation process that still require attention. One such aspect is in the area of multisectoral collaboration. Multisectoral action is key to any successful rollout of an injury prevention strategy (14). Although the SoLiD Project actively engaged the private sector in Bangladesh to facilitate local production and supply of the intervention package, there is still potential for involving other sectors. For example, an effective drowning prevention measure may cut across the transportation sector to minimize water transport mishaps; the education sector to serve as an intervention platform for reaching the population most at risk; and the health sector to promote family and community health.

Exploring these potential pathways for multisectoral collaboration is important for optimizing available resources in Bangladesh to reduce the burden of drowning. The current support from Bloomberg Philanthropies is a critical investment in the field of drowning prevention and will facilitate the initial take-off of the SoLiD implementation process and also stimulate other donors to explore this field. However, to foster long-term sustainability, it is imperative that the communities at risk gain ownership of the implementation process. A more direct approach for accomplishing the sustainability goal may be to integrate the project aims into national policies and plans and use the existing public resources to advance these objectives for the common good (14). This approach requires significant political capital even after documenting the large-scale effectiveness of the interventions. The SoLiD Project seeks to achieve this by active involvement of the recipient communities and engaging with the public sector; its long-term sustainability is predicated on a significant pool of the population at risk adopting (and observing the benefit of) the interventions, thereby creating a demand for the interventions. For this community-based and market-driven approach to be successful, the local industry must possess the capacity to meet the potential future demand for interventions.

The baseline and surveillance data from the Project will provide more accurate estimates of the burden of injuries in Bangladesh and supplement the world's data pool with high-quality, injury-related information in resource-poor settings. These types of data are lacking and affect the quality of existing global estimates. The SoLiD Project is an important opportunity for global estimates to be revisited. However, the data system for SoLiD is not designed for all purposes and, for example, does not track disability over time and does not collect health facility-based information.

The potential benefits of this project may extend beyond reducing the burden of childhood drowning in Bangladesh. For instance, implementation of the playpen intervention will require local production of wooden and plastic playpens to ensure adequate supply while controlling costs. This venture will create employment opportunities among the local communities where the Project will be implemented (12). Similarly, the Project will provide income-generation opportunities for local women who will be involved in providing key services at the crèches (12). The children enrolled in the study are also likely to benefit from the educational activities in the crèche; this will enhance their cognitive development and the full attainment of their academic potential (12).

Global knowledge exists on how to prevent drowning-related deaths in LMICs. The key to reducing deaths is the effective implementation of known and new interventions. This paper is expected to stimulate a global dialogue on how to do this at scale.

## ACKNOWLEDGEMENTS

The authors would like to acknowledge Dr. D.M. Emdadul Hoque and Ms Shumona Sharmin of the Centre for Child and Adolescent Health and the Geographic Information System Unit at icddr,b for the map used in Figure 1. This study is supported by Bloomberg Philanthropies, New York.

**Conflict of interest:** The authors declare that they have no competing interests.
